# 60 fs, 1030 nm FEL pump–probe laser based on a multi-pass post-compressed Yb:YAG source

**DOI:** 10.1107/S1600577520015052

**Published:** 2021-01-01

**Authors:** Anne-Lise Viotti, Skirmantas Alisauskas, Ammar Bin Wahid, Prannay Balla, Nora Schirmel, Bastian Manschwetus, Ingmar Hartl, Christoph M. Heyl

**Affiliations:** a Deutsches Elektronen-Synchrotron DESY, Notkestrasse 85, 22607 Hamburg, Germany; b Department of Physics, Lund University, PO Box 118, SE-221 00 Lund, Sweden; c Helmholtz-Institute Jena, Fröbelstieg 3, 07743 Jena, Germany

**Keywords:** X-ray FEL, pump–probe, ultrafast lasers, burst-mode lasers, nonlinear post-compression, multi-pass cells

## Abstract

This paper reports on nonlinear spectral broadening of 1.1 ps pulses in a gas-filled multi-pass cell to generate sub-100 fs optical pulses at 1030 nm and 515 nm at pulse energies of 0.8 mJ and 225 µJ, respectively, for pump–probe experiments at the free-electron laser FLASH. Combining a 100 kHz Yb:YAG laser with 180 W in-burst average power and a post-compression platform enables reaching simultaneously high average powers and short pulse durations for high-repetition-rate FEL pump–probe experiments.

## Abstract 2

This paper describes a short-pulse laser source based on post-compression via multi-pass spectral broadening of a 1030 nm burst-mode laser employed for FEL pump–probe experiments.

## 1. Introduction   

The bright pulses of X-ray free-electron lasers (FELs) allow ultrafast measurements in the extreme ultraviolet (XUV) and soft X-ray regimes with femtosecond (fs) time resolution, providing exciting opportunities to study ultrafast dynamics within multiple research fields including physics, chemistry and biology (Abramczyk, 2005 ▸; Zewail, 2000 ▸; Seddon *et al.*, 2017 ▸). Such experiments are often carried out using so-called pump–probe techniques: a first light pulse (in the infrared, visible, ultraviolet or X-ray regime) is used to initiate a dynamical process such as, for example, a chemical reaction or a phase transition in the sample under study, followed by a second pulse at a well defined time-delay to probe the system response. Two essential tools are typically required: an X-ray FEL and an optical pump–probe laser tailored for specific scientific experimental needs. Both should have the same pulse pattern and a controllable time-delay with femtosecond precision.

FLASH is the first worldwide high-repetition-rate XUV and soft X-ray FEL facility, which enables multi-user experiments via two simultaneously operating FEL beamlines FLASH1 and FLASH2 (Faatz *et al.*, 2016 ▸; Plönjes *et al.*, 2016 ▸). FLASH2 FEL provides pulse durations as short as 10 fs and pulse energies up to 1 mJ at 30 nm to 4 nm (about 40 to 300 eV) and is equipped with a highly flexible optical pump–probe laser. Matched to the pulse structure of the FEL, this pump–probe laser based on optical parametric chirped pulse amplification (OPCPA) emits ultra-short pulses in 10 Hz bursts (<800 µs) with tunable pulse duration (15 to 100 fs), wavelength (700–900 nm), polarization and pulse energy (200 µJ at the experimental user end-stations) at 100 kHz intra-burst repetition rate (Lang *et al.*, 2019 ▸; Alisauskas *et al.*, 2019 ▸). The OPCPA is pumped by a powerful Yb:YAG laser (10% pump-to-OPCPA signal conversion efficiency). The wavelength range of this OPCPA system can easily be extended, for example by utilizing harmonic frequency conversion or parametric amplification. In order to meet the experimental demands on a broad range of optical pump–probe wavelengths, a spectral coverage extending all the way from THz frequencies into the UV is envisioned within the FLASH2020+ upgrades (Beye & Klumpp, 2020 ▸).

As an alternative to converting the output of a complex OPCPA system into other wavelength regimes, a direct conversion of high-power ytterbium (Yb) pump lasers appears attractive. Yb-based laser systems can easily deliver hundreds of Watts at MHz repetition rates (Müller *et al.*, 2018 ▸; Schmidt *et al.*, 2017 ▸) but with relatively long pulse durations of a few hundreds of femtoseconds (Yb:fiber) up to 1 ps (Yb:YAG) due to gain bandwidth limitations. In order to combine the high average power of Yb-based laser systems with short pulse durations required for FEL pump–probe experiments, external pulse post-compression schemes can be exploited. Such methods rely on nonlinear spectral broadening by self-phase modulation (SPM) in a Kerr medium, followed by chirp compensation (Tomlinson *et al.*, 1984 ▸). Various spectral broadening approaches have been demonstrated employing, for example, solid-core fibers (Shank *et al.*, 1982 ▸), gas-filled hollow-core capillaries (Nisoli *et al.*, 1996 ▸) or hollow-core type fibers (Russell *et al.*, 2018 ▸, and references therein). However, the spectral broadening performance required for the compression of high-power Yb:YAG pump lasers delivering pulses in the 1 ps multi-mJ range down to the sub-100 fs regime reaches the parameter limits accessible via common post-compression methods. While hollow-core capillary-based post-compression schemes support mJ-class pulses and large compression ratios, setup lengths easily reach practical limits at these parameters (Nagy *et al.*, 2019 ▸). Recently, a new post-compression method was developed, employing spectral broadening in a Herriott-type multi-pass cell (MPC) (Herriott *et al.*, 1964 ▸; Schulte *et al.*, 2016 ▸). In particular, gas-filled MPC provides large compression ratios and efficiencies above 90% while allowing compact setups.

Here, a simple laser scheme based on multi-pass spectral broadening of picosecond pulses is implemented to generate sub-100 fs pulses with high average powers in order to cover the wavelength range around 1 µm and its second harmonic. Building on very recent results (Balla *et al.*, 2020 ▸) we develop an 80 W, 100 kHz (in-burst parameters at the experimental end-station), 60 fs source and demonstrate its long-term operation in preparation for upcoming FEL pump–probe experiments.

The paper is structured as follows: Section 2 presents the experimental setup, Section 3 describes the main results, Section 4 addresses long-term measurements for stable MPC operation within the FEL facility and Section 5 summarizes our findings.

## 2. Experimental setup   

**Figure 1:**
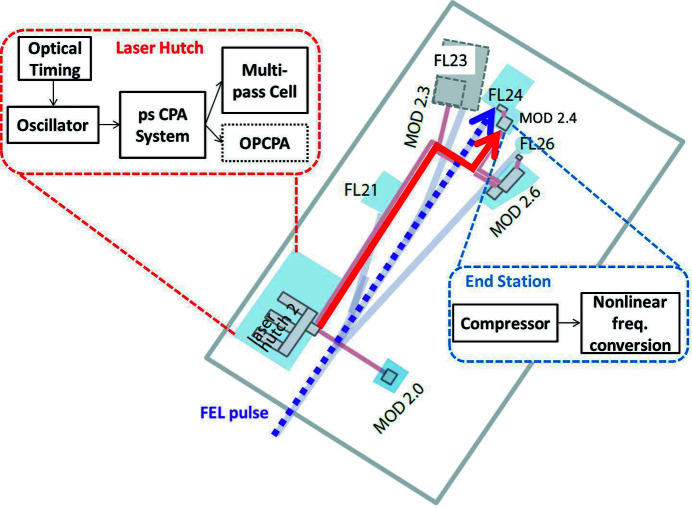
FLASH2 beamline overview with the locations of the laser hutch and the experimental end-stations as designed according to the FLASH2020+ upgrade. The insets show the schematic layout of the experiment, including oscillator, amplifier and MPC in the laser hutch and compressor as well as nonlinear frequency conversion unit at the user end-station. The solid red and dashed purple arrows represent laser and FEL pulse delivery, respectively. The sketch is adapted from Beye & Klumpp (2020 ▸). The gray out end-station FL23 is planned to be built in the near future.

The FLASH2 pump–probe laser is located in a closed hutch (see Fig. 1 ▸). The optical pulses used for FEL pump–probe experiments are transported to the FLASH2 modular optical delivery setups (MODs) close to the user experiments via a 40 m-long relay-imaging beam transport line with high temporal and spatial stability. The FLASH2 pump–probe laser consists of an oscillator centered at 1030 nm, which is synchronized to the FLASH FEL master oscillator via a pulsed optical timing distribution system using length-stabilized fibers (Schulz *et al.*, 2015 ▸). This optical link includes a precisely variable time-delay for pump–probe experiments. The oscillator output seeds two chirped pulse amplifiers (CPA): a sub-500 fs Yb-fiber amplifier used for white light generation for the OPCPA and a 1 ps Yb:YAG Innoslab amplifier (Russbueldt *et al.*, 2009 ▸) used as the OPCPA pump source. For our experiment, we employ the output of the ps CPA, which delivers 400 W in burst (4 mJ single pulse energy at 100 kHz) and adjustable pulse energies depending on the repetition rate of the laser, and post-compress this laser using a MPC-based compression scheme (Balla *et al.*, 2020 ▸). In the present setup, we use 180 W (1.8 mJ single pulse energy) at 1030 nm, with a pulse duration of 1.1 ps (full width at half-maximum, FWHM) in a 10 Hz burst with an intra-burst repetition rate of 100 kHz. This results in a total average power of 1.4 W. The MPC input beam has an M^2^ of 1.2 × 1.1.

**Figure 2:**
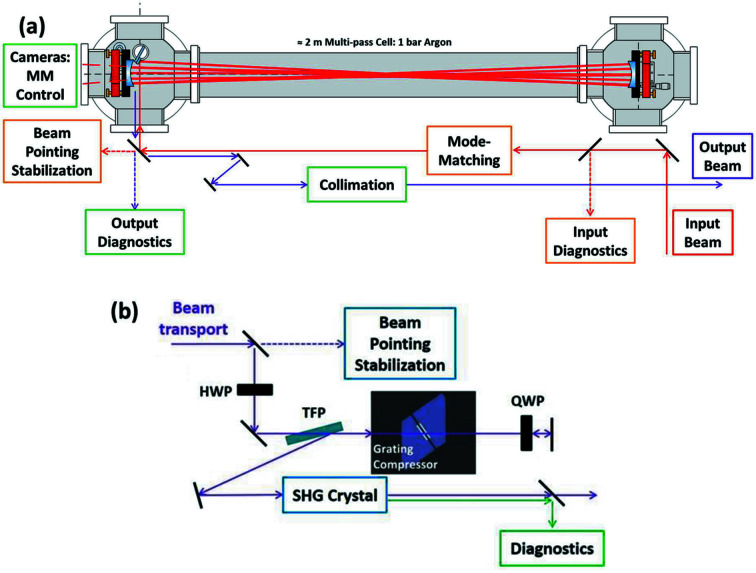
Optical setups for (*a*) the MPC in the laser hutch and (*b*) the compression and second harmonic generation (SHG) at the user end-station. MM: for mode-matching; HWP: half wave-plate; QWP: quarter wave-plate; TFP: thin-film polarizer.

As outlined in Fig. 1 ▸, the setup in the laser hutch includes the OPCPA pump laser system, as well as the MPC. The actual compression and subsequent nonlinear frequency conversion are performed at MOD2.4, after transport of the uncompressed pulses in vacuum in order to avoid any detrimental effects caused by B-integral over the 40 m propagation distance. The nonlinear frequency conversion scheme reported in this paper is second-harmonic generation (SHG), developed for a specific user experiment requiring sub-100 fs, >100 µJ pulses at 515 nm. Detailed setups for both the MPC and the user end-station are shown in Fig. 2 ▸.

The experimental setup benefits from beam pointing stabilization units at critical locations to guarantee stable operation of the system: at the input of the MPC and at the input and output of the 40 m beam transport unit. Additionally, the laser, the spectral broadening units and the MODs are equipped with various beam diagnostics.

The MPC employs gas as a nonlinear medium for spectral broadening as it allows an easy tuning of the spectral bandwidth by varying the gas pressure and thus adjusting the nonlinearity. Moreover, as opposed to bulk nonlinear media, gas is immune to damage. The near-concentric cell shown in Fig. 2 ▸(*a*) is filled with 1 bar of argon and consists of two dielectric broadband concave mirrors with radius of curvature of 1 m and 100 mm diameter. The MPC is operated with 18 roundtrips and the laser beam is mode-matched to the MPC eigenmode via a mode-matching lens telescope. In- and out-coupling of the cell is done via the same mirror but with a small angle between in- and out-going beams, which allows spatial separation of the beams after some propagation distance. The output beam is then collimated with a spherical mirror followed by a mirror telescope. The setup at the endstation is shown in Fig. 2 ▸(*b*). It consists of a compressor with four passes though transmission gratings (1000 lines mm^−1^). The compressor design allows for easy variation of the grating distance, *i.e.* adjustment of the chirp of the compressed pulses, without changing drastically the timing relative to the FEL pulse, as opposed to a chirped mirrors compressor where the number of bounces and thus the optical path length has to be adjusted. Out-coupling of the compressor is performed via reflection off a broadband thin film polarizer operating at an angle of 70°, thanks to the polarization change introduced by a quarter wave-plate. It should be noted that our gratings are polarization-insensitive. The beam passes through a wave plate located in front of the TFP in order to be able to control the pulse energy. After the compressor, the beam is sent through different SHG lithium triborate (LBO) crystals with variable thickness allowing optimizing SHG bandwidth, conversion efficiency and beam quality. The beam is not focused into the SHG crystal to avoid saturation effects and to maximize beam quality while accepting reduced conversion efficiency compared with SHG driven with a focused beam.

## 3. Results   

The main laser output parameters are summarized in Table 1 ▸. In particular, M^2^ measurements are performed at specific locations in the experimental setup to monitor eventual beam degradation due to B-integral effects, for example in the cell, in the 40 m beam transport, or after the compressor where short fs pulses might self-focus in air. The M^2^ values are shown in the last row of Table 1 ▸.

**Figure 3:**
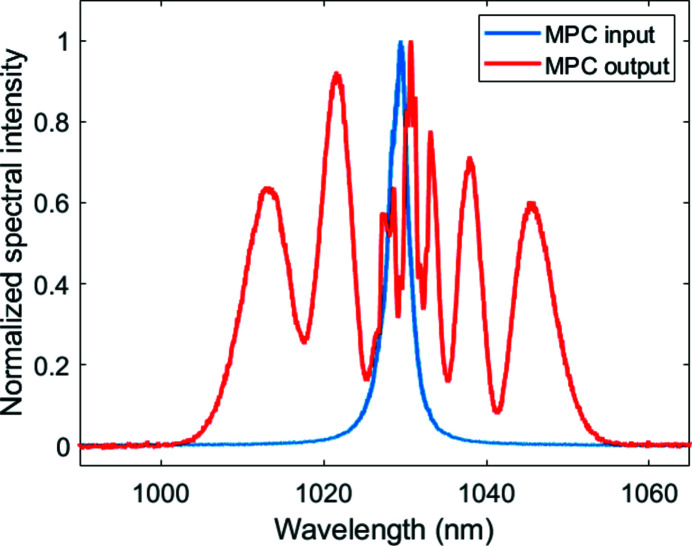
Measured input (blue) and output (red) spectra of the MPC.

The output of the MPC is characterized in terms of output pulse energy (with a calibrated photodiode), spectrum and pulse duration. Fig. 3 ▸ shows a typical broadened spectrum with the common SPM ripple pattern.

**Figure 4:**
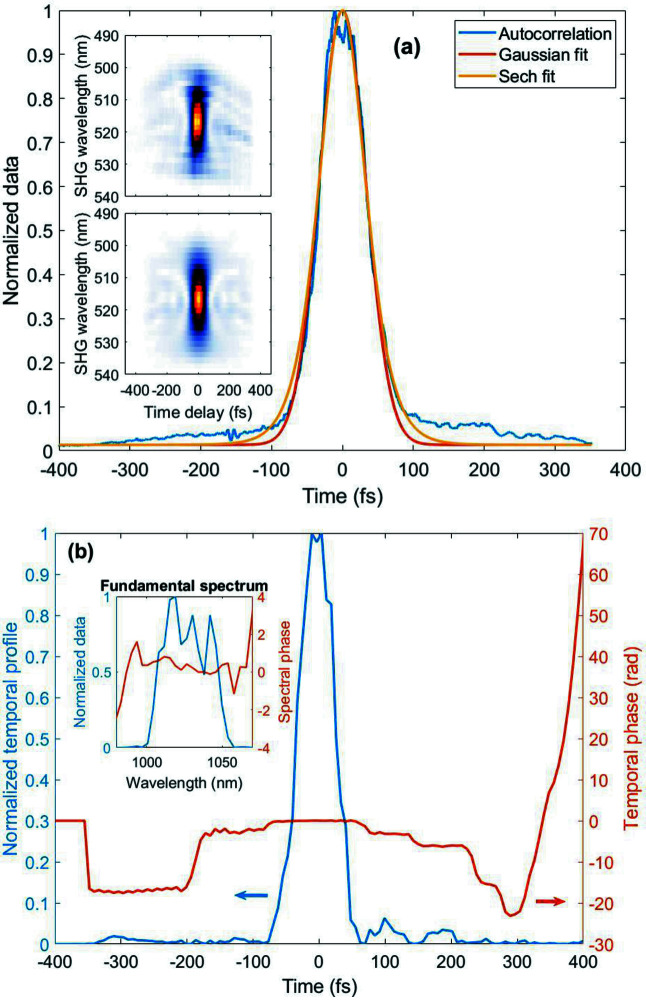
(*a*) Autocorrelation measurement of the MPC output pulses. The insets show the measured and retrieved FROG traces. (*b*) Retrieved temporal profile and phase as well as retrieved fundamental spectrum and spectral phase at 1030 nm (inset). The spectral resolution is too low here to fully resolve the SPM ripples of the spectrum at the output of the MPC, limited by the resolution of the FROG unit (Femtoeasy FROG). The integration time for the measurement was 100 µs in the central part of the burst.

The Fourier limit of the MPC output spectrum shown in Fig. 3 ▸ is 60 fs FWHM. The compressed pulse duration after the grating compressor at the end-station is measured via single-shot SHG frequency resolved optical gating (SHG-FROG) with 100 µs integration time (*e.g.* integrating 10 pulses in the central part of the burst). The measured and retrieved FROG traces are shown as insets in Fig. 4 ▸(*a*), with a retrieval error of 4%.The FROG traces show a temporal background pedestal which extends to the duration of the pump pulse. Fig. 4 ▸(*b*) shows the retrieved temporal pulse profile, with a duration of 61 fs FWHM, well matching the data of Fig. 3 ▸. The transmission of the compressor setup is about 54% yielding a maximized output pulse energy of 0.8 mJ. It should be noted that the MPC configuration used here is not optimized for maximum compression ratio or maximum pulse energy, but rather adapted to meet the experimental requirements with a highly stable and reproducible setup, as opposed to the work presented by Balla *et al.* (2020 ▸), targeting shortest pulse durations.

**Figure 5:**
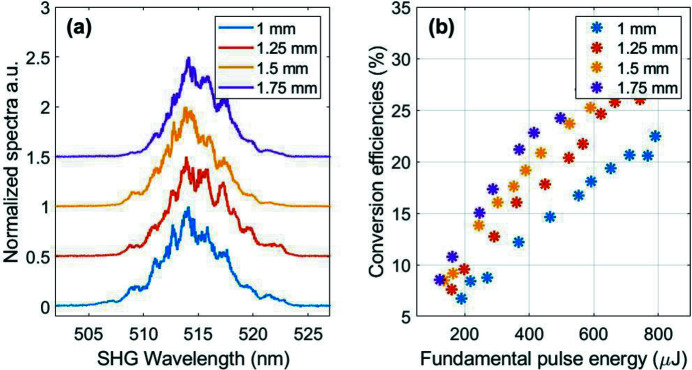
(*a*) SHG spectra centered at 515 nm for different LBO crystal lengths. The spectra are vertically displaced for clarity. (*b*) SHG conversion efficiencies for the different crystals.

Fig. 5 ▸ shows SHG spectra and corresponding conversion efficiencies for several LBO crystals. The nominal SHG beam size was 5.8 mm × 6.3 mm.

For the user experiment, the spectrally broadened and temporally compressed pulses at 1030 nm are frequency doubled [see Fig. 2 ▸(*b*)] in an LBO crystal. As shown in Fig. 5 ▸(*b*), the SHG conversion efficiencies do not flatten to a plateau but start to roll off at the maximum input pulse energy used. More than 30% conversion efficiency can be reached without focusing the beam into the crystal, which corresponds to an output SHG pulse energy of 225 µJ. The measured FWHM bandwidths of the spectra in Fig. 5 ▸(*a*) are 5.8 nm, 5.5 nm, 4.7 nm and 5.1 nm for crystal thicknesses of 1 mm, 1.25 mm, 1.5 mm and 1.75 mm, respectively. Those values correspond to Fourier limits which are well below 100 fs FWHM. The spectral widths are smaller than the width of the corresponding SHG spectrum measured with the single-shot SHG FROG, which employs a thinner crystal.

**Figure 6:**
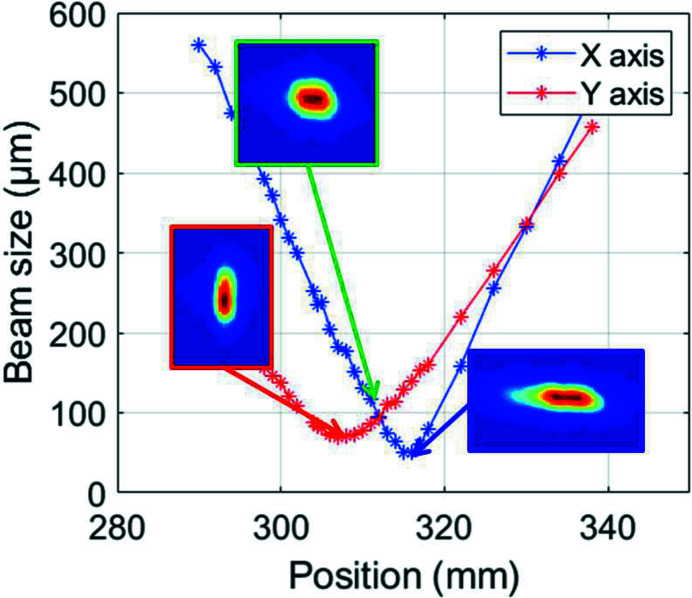
M^2^ measurements for the SHG beam using the 1 mm LBO crystal. The three beam profile insets show elongated beam profiles along the horizontal and vertical dimensions as well as the beam at the crossing of both caustics. The measured M^2^ value for the SHG beam is 2.03 × 1.63.

The SHG output is further characterized via M^2^ measurements. Results for the 1 mm-long LBO are presented in Fig. 6 ▸, together with a few beam profiles measured at a few example locations. The beam is clearly astigmatic, which we attribute mainly to beam asymmetries of the pump laser beam caused by the cylindrical focusing and amplification geometry of the Yb:YAG amplifier.

While the temporal properties of the 515 nm pulses are not measured, the measured pulse durations below 100 fs of the 1030 nm beam indicate a SHG pulse duration under 100 fs.

## 4. Long-term stability   

**Figure 7:**
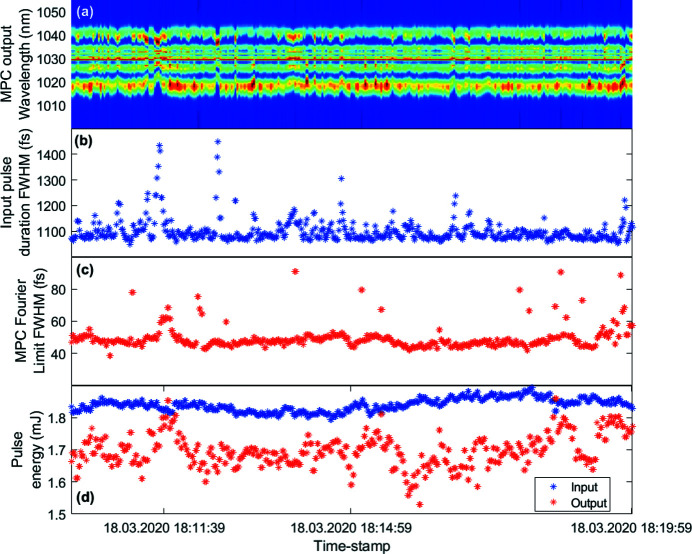
(*a*) Broadened output spectrum of the MPC. (*b*) MPC input pulse duration measured by a single-shot autocorrelator with 30 µs integration time, *i.e.* 3 pulses in the center of the burst. (*c*) FWHM Fourier limit of the MPC output spectra. (*d*) Pulse energies for MPC input and output. All data are plotted for the same 10 min time-stamps. Reading error of the photodiode server used to calibrate the pulse energy explains the few higher red points with respect to the blue data points in (*d*).

The performance of the complete laser system is recorded over the course of several days in order to evaluate the overall system stability. As summarized already in Table 1 ▸, the MPC output has a pulse energy fluctuation below 4% RMS. The MPC output spectrum is also monitored to verify long-term stability and in particular a possible influence of peak intensity variations in the cell. This is important as the Yb:YAG CPA system exhibits pulse length variations, in the range from about 1.05 ps up to 1.325 ps for a few instances. The corresponding standard deviation with respect to the mean value of 1.095 ps is 36 fs RMS (3.3% RMS), *i.e.* the peak values at durations >1.3 ps have relatively rare occurrences. As an example data set, Fig. 7 ▸ displays a 10 min interval of a 36 h data set recorded at 10 Hz. While the spectrum is clearly ‘breathing’, the Fourier-limited bandwidth stays below 100 fs at all times. Figs. 7 ▸(*b*) and 7(*d*) show input pulse duration and MPC in- and output pulse energies recorded at the same time stamps as the spectral data.

Figs. 7 ▸(*a*) and 7(*b*) clearly show that an increased input pulse duration correlates with a decreased bandwidth of the generated MPC output spectrum. Nevertheless, the output pulses of the MPC are kept below 100 fs for input pulse energies above 1.5 mJ. While the displayed data set is limited to 10 min, all parameters show a similar performance and prove good long-term stability over the 36 h recording time frame.

**Figure 8:**
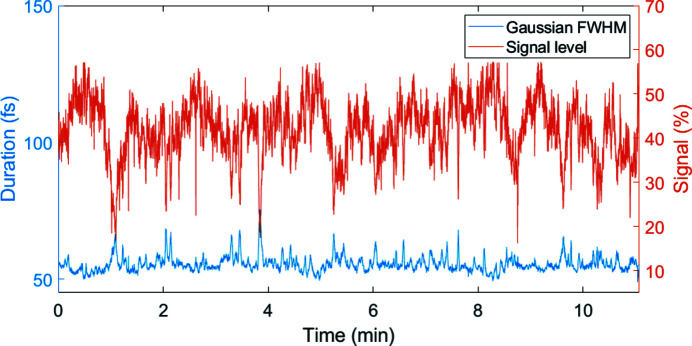
History of the MPC output pulse duration (blue) of the compressed pulses measured using a single-shot FROG with 100 µs integration time in the central part of the burst at the MOD2.4. The signal level (orange) is expressed in percent, normalized to the saturation signal level of the device and is proportional to intensity fluctuations.

The compressed output pulse duration of the MPC is also logged after the grating compressor at the user end-station with a single-shot FROG. The reconstructed output pulse duration (FWHM) is presented in Fig. 8 ▸, together with the device input signal level. The pulse duration is on average 55 fs, with a standard deviation of 2.8 fs (5.1% RMS). The visible spikes indicate an increased pulse duration, which are most likely due to an insufficient signal level for the FROG measurement.

**Figure 9:**
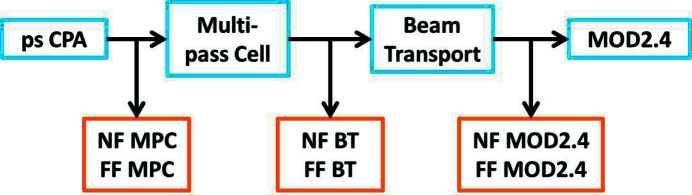
Schematic illustration of the locations of the beam pointing stabilization units and the mode-matching camera control. ‘NF’ stands for near-field, ‘FF’ for far-field and ‘BT’ for beam transport.

In addition to the spectral and temporal characterization, the beam pointing stability is also monitored over 36 h. Different sets of cameras are installed along the beam path, as illustrated in Fig. 9 ▸. Three beam pointing stabilization units, consisting of both near- and far-field beam imaging (collimated and focused beam, respectively) are located at the input to the MPC (labeled ‘MPC’), the entrance to the 40 m beam transport (‘BT’), as well as at the MOD (‘MOD2.4’). Each beam pointing stabilization camera set is connected via a digital control loop to two mirrors in the optical beam path for active pointing drift stabilization. In addition, a set of two cameras is positioned behind one of the MPC mirrors, as shown in Fig. 2 ▸(*a*). These cameras are monitoring two beam spots diametrically opposed in the typical circular (or elliptical) pattern that the beam forms at the mirrors of a Herriott-type MPC. The eventual position and size variation of these spots can help to identify mode-matching and beam size drifts and thus prevent damage of the cavity mirrors. As mode-matching requires identical beam sizes for all beam spots at the MPC mirrors, mode-matching drifts can easily be identified by beam size monitoring.

The cameras for near-field (‘NF’) and far-field (‘FF’) monitoring have a pixel size of 11 µm and 3.75 µm, respectively. The results are summarized in Table 2 ▸, where the main parameters of interest are listed.

All beam pointing instabilities are relatively small at the three experimental locations over 36 h. The beam transport does not increase pointing instability. Stable beam diameters recorded using the Herriott pattern monitor cameras indicate that drifts of the beam mode-matching into the MPC are also very small.

**Figure 10:**
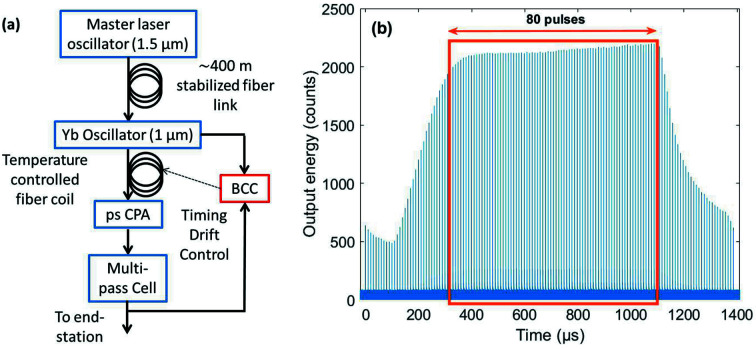
(*a*) Schematic overview of the timing drift control setup based on balanced cross-correlation. (*b*) Output burst of the MPC at 1030 nm showing the relatively flat part of the burst that was employed for timing jitter measurement (orange rectangle corresponding to 80 pulses).

The pump chain is seeded by a low noise Yb laser oscillator, which is synchronized to FLASH’s master oscillator pulsed optical reference delivered by a length stabilized fiber link (Schulz *et al.*, 2015 ▸; Schirmel *et al.*, 2019 ▸). Part of the laser oscillator output is cross-correlated with the output of the MPC in a balanced cross-correlator (b.c.c.) for active timing drift stabilization, which is performed via a temperature-controlled fiber spool located between the oscillator and the ps CPA unit. An overview of the timing drift setup is shown in Fig. 10 ▸(*a*).

**Figure 11:**
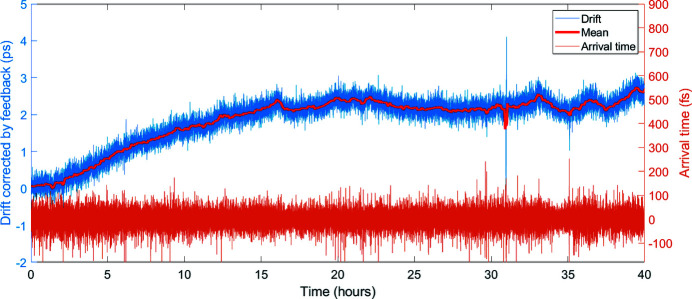
Corrected drift by temperature feedback (blue) and arrival time (orange) for the MPC output burst recorded over a 40 h time span with a b.c.c. The sub-100 fs RMS arrival time jitter is an upper limit value, estimated taking into account calibration uncertainties for the measurement.

In a very preliminary study, the burst-to-burst jitter was measured for 80 pulses located in the flat part of the MPC output burst [see Fig. 10 ▸(*b*)]. One should note that the total path length in the MPC is 72 m. A sub-100 fs RMS arrival time jitter was recorded at 1 Hz over 40 h. Minimization of the timing jitter and full characterization of intra-burst jitter were not performed but are planned in the near future. Moreover, the introduced temperature change of the fiber coil was monitored over the same time span. From this, the drift corrected by the feedback can be derived (see Fig. 11 ▸). Logged data showed that the entire amplifier chain together with the MPC exhibit a 2.7 ps drift over 40 h. Both arrival time and drift data are presented in Fig. 11 ▸, for the same time span.

## 5. Conclusions and outlook   

In summary, we report a sub-100 fs laser source based on nonlinear multi-pass spectral broadening of a high average power, high repetition rate, ps laser system. The ps laser pulses at 1030 nm are spectrally broadened and compressed to pulse durations around 60 fs with pulse energies in the mJ range. The transmission of the MPC exceeds 85% and operation of the cell is stable over the course of several days, making the setup suitable for FEL user experiments. For pump–probe experiments requiring sub-100 fs pulses at 515 nm, the compressed mJ pulses are frequency doubled in LBO crystals with up to 30% conversion efficiency. The resulting SHG pulses at 515 nm reach pulse energies well above 100 µJ, with sub-100 fs duration and sufficient beam quality (M^2^ ≤ 2). The long-term measurements show good stability regarding the output spectrum of the MPC, output pulse energy, as well as beam pointing and beam size. With a first implementation of a slow timing drift feedback system, a sub-100 fs RMS arrival time jitter could be measured between the oscillator and the MPC output. Future work will focus on intra-burst characterization for better understanding of the pulse dynamics inside the burst itself.

The here presented work adds sub-100 fs, mJ-class laser pulses at 1 µm and subsequent harmonics, to the FLASH2 pump–probe laser capabilities. This way, our work enables new parameter regimes and offers additional opportunities for further wavelength conversion schemes pumped with the output of the MPC setup. Ongoing developments target, for example, pumping of an optical parametric amplifier (OPA) using the MPC output. In addition, a second spectral broadening stage as presented by Balla *et al.* (2020 ▸), can provide few-cycle pulses to enable further parameter tunability of FLASH2 laser system.

## Figures and Tables

**Table 1:** Summary of the key laser parameters The energy stability was logged over 36 h. For the MPC output, both M^2^ values inside the laser hutch and after the compressor at the MOD2.4 are shown. The pulse duration mentioned for the MPC output corresponds to the cell parameters employed in this paper. A large range covering at least 30 to 100 fs can easily be reached by tuning the gas pressure and/or optimizing the number of roundtrips in the cavity as well as the compressor settings.

Parameters	Laser	MPC output
Pulse energy (single pulse, mJ)	1.79	1.68
Pulse duration (fs)	∼1100	∼60
Central wavelength (nm)	1030	1030
Intra-burst repetition rate (kHz)	100	100
Burst repetition rate (Hz)	10	10
Energy stability (% RMS)	1.7	3.5
M^2^ (*X*, *Y*)	1.25, 1.15	1.29, 1.24 (laser hutch)
		1.65, 1.74 (MOD2.4, compressed)

**Table 2:** Beam pointing stability over 36 h for near-field (‘NF’) and far-field (‘FF’) data, variations in percent of the beam diameter for NF and fluctuations in percent of the beam divergence angle for FF, thanks to M^2^ measurements

	MPC input	Beam transport	MOD2.4
*X*_NF_ fluctuations (% of NF diameter)	2.7	3.1	2.6
*Y*_NF_ fluctuations (% of NF diameter)	1.8	3.1	2.5
*X*_FF_ fluctuations (% of beam divergence)	0.5	0.6	1.6
*Y*_FF_ fluctuations (% of beam divergence)	0.7	0.5	0.4
